# Self-Assembly of Microscopic Rods Due to Depletion Interaction

**DOI:** 10.3390/e22101114

**Published:** 2020-10-01

**Authors:** Carles Calero, Ignacio Pagonabarraga

**Affiliations:** 1 Departament de Física de la Matèria Condensada, Universitat de Barcelona, 08028 Barcelona, Spain; ipagonabarraga@ub.edu; 2 Institut de Nanociència i Nanotecnologia, Universitat de Barcelona, 08028 Barcelona, Spain; 3 Universitat de Barcelona Institute of Complex Systems (UBICS), Universitat de Barcelona, 08028 Barcelona, Spain; 4 CECAM, École Polytechnique Fédérale de Lausanne, Batochime, Avenue Forel 2, 1015 Lausanne, Switzerland

**Keywords:** depletion forces, self-assembly, Langevin dynamics

## Abstract

In this article, using numerical simulations we investigate the self-assembly of rod-like particles in suspension due to depletion forces which naturally emerge due to the presence of smaller spherical depletant particles. We characterize the type of clusters that are formed and the evolution of aggregation departing from a random initial configuration. We show that eventually the system reaches a thermodynamic equilibrium state in which the aggregates break and reform dynamically. We investigate the equilibrium state of aggregation, which exhibits a strong dependence on depletant concentration. In addition, we provide a simple thermodynamic model inspired on the theory of self-assembly of amphiphilic molecules which allows us to understand qualitatively the equilibrium aggregate size distributions that we obtain in simulation.

## 1. Introduction

Due to their entropic origin, depletion forces are ubiquitous in complex fluids. They originate in solutions with high concentrations of microscopic particles of at least two different sizes. Above certain threshold concentrations of the different species, the larger particles tend to aggregate to increase the entropy of the system and thus minimize its free energy. This phenomenon can be seen as an effective interaction between the larger particles mediated by the smaller ones, as formulated by Asakura and Oosawa in their seminal work [1]. Depletion interactions are of paramount importance to understand colloidal stability, as they can cause flocculation [2]. In addition, depletion forces are thought to be a significant contributor in some biological phenomena of special relevance [3], such as the formation of aggregates of erythrocytes in blood [4].

Since the first theoretical account of depletion forces was given by Asakura and Oosawa [1], much effort has been devoted to determine, both theoretically [5,6,7,8,9] and experimentally [10,11], the basic properties of the depletion interaction in a variety of colloidal systems. To test the theoretical picture and gain further insight, computer simulations have been used to investigate the dependence of depletion forces on distance, depletant concentration, and the geometry of the colloidal particles [12,13,14,15]. In addition, there is also a large amount of work devoted to the phenomenological description of the structures generated by depletion in colloidal dispersions [16,17,18,19].

The interaction due to depletion can be markedly anisotropic depending on the shape of the interacting colloids. A number of different naturally occurring and custom-made shapes have been investigated that self-assemble due to depletion forces into a rich variety of complex structures [17,18,20,21]. In fact, due to its anisotropy, the effect of depletion has been proposed as a possible driving force to direct the self-assembly of colloids into predesigned structures in the quest to synthesize new functional materials [16,17,18,20,22].

In this article, we investigate using numerical simulations the aggregation of rod-like particles due to depletion forces. In contrast to most simulation studies where the depletion interaction is introduced via effective potentials between colloidal particles, in our approach we simulate the dynamics of both the rod-like particles and smaller spherical depletant particles, which naturally induce the emergence of depletion forces. Departing from a random configuration, we solve the evolution of the system until the average aggregate size (and thermodynamic quantities such as rotational and translational kinetic energies) reach a stationary state which we identify with thermodynamic equilibrium. We characterize the equilibrium size distribution of the aggregates and the dependence of aggregation on the concentration of depletant. In addition, we propose a new theoretical approach in the discussion of depletion forces inspired on the theory of self-assembly of amphiphilic molecules, which allows us to understand qualitatively the equilibrium distributions that we obtain in simulation.

## 2. Computer Simulations

**Figure 1:**
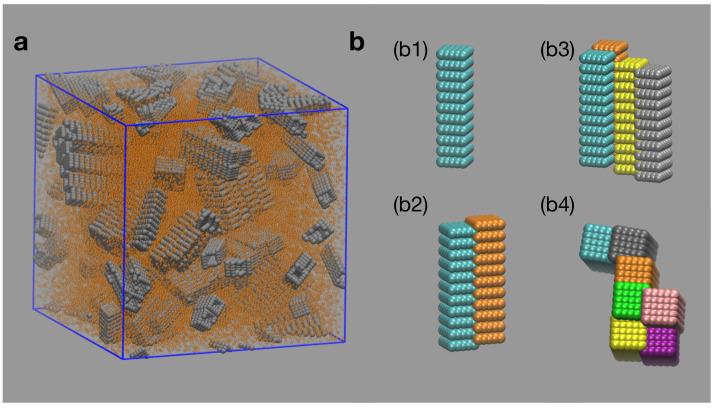
(**a**) Snapshot of simulated system. The spherical beads forming the rods are represented in gray. Depletant particles are represented by orange beads. (**b**) Frequent assemblies observed in simulations: (**b1**) monomer; (**b2**) pair of side-to-side assembled rods; (**b3**) bundle of four rods with side-to-side aggregation; (**b4**) top view of large bundle, formed exclusively by side-to-side bonds. All rods are identical, different colors were used for visualization purposes.

We performed computer simulations of a system of long rods in a solution of smaller depletant spherical particles in contact with a thermal bath. The rods, of length *L* and square section of side *a*, are modeled with the help of a group of spherical beads which collectively behave as a rigid body [23] (see Figure 1). The spherical beads forming a rod do not interact with one another, but they interact with spherical beads belonging to other rods and with spherical depletant particles. The interactions among all the spherical beads in the simulation are purely repulsive, modeled through the Weeks–Chandler–Andersen (WCA) potential

$$V\left(r\right)=\left\{\begin{array}{c} 4\epsilon_{ij}\left[{\left(\frac{\sigma_{ij}}{r}\right)}^{12}-{\left(\frac{\sigma_{ij}}{r}\right)}^{6}\right]+\epsilon_{ij}\;,\;r<2^{1/6}\sigma_{ij}\\ 0\;,\;r\geq 2^{1/6}\sigma_{ij}\;, \end{array}\right.$$

Here, *r* is the distance between particles *i* and *j*, $\epsilon_{ij}$ defines the energy, and $\sigma_{ij}$ is the range of the interaction. In the cases considered, we have chosen $\epsilon_{ij}\equiv \epsilon =1$ for all interactions, $\sigma_{11}\equiv \sigma =1$ for the interaction between the particles that form the rods, $\sigma_{22}=1.2$ for interactions between depletant particles, and $\sigma_{12}=1.1$ for interactions between disc and depletant particles following the Lorentz–Bertheloz rule. The values of $\sigma_{11}$ and $\sigma_{22}$ are a measure of the diameter of the beads forming the rods and the depletant particles, respectively. The WCA potential is a continuous potential which generates purely repulsive interactions. This allows us to discard direct particle interactions as a source of colloidal aggregation. Periodic boundary conditions are applied in all three directions.

The dynamics of rods and depletant particles is solved by integrating numerically the Langevin stochastic equations of motion. In addition to particle–particle interactions, these equations consider the effect of the medium through a viscous dragging force opposing the motion of particles and a stochastic force corresponding to thermal noise. In our treatment, we have neglected the effect of sedimentation assuming that the particles considered are neutrally buoyant. All simulations were performed using the simulation package HOOMD-blue 2.2.3 [24,25] running on Tesla P100 GPUs (See Supplementary Materials).

We report results obtained by considering systems with 125 rods (each one composed of 194 beads) of length L=20σ and square section of side a=4.5σ in a cubic simulation box of side $L_{box}=80\sigma$ (see Figure 1). We consider four different cases with different concentrations of depletant particles, $c_{D}=0.088\sigma^{-3},0.098\sigma^{-3},0.118\sigma^{-3},0.129\sigma^{-3}$. The number of particles in the system varies from 69,735 to 90,375 depending on the depletant concentration considered. The system is prepared departing from a lattice configuration with all rods and depletant particles in a large simulation box to avoid overlaps. Next, a short simulation is performed in which the dimensions of the simulation box are reduced as time advances until the desired density of particles is reached. From that configuration we perform Langevin simulations with fixed volume, temperature, and number of particles to investigate the formation of aggregates due to the emergent depletion interaction. The temperature of the system is set to $k_{B}T=1$, $k_{B}$ is the Boltzmann constant, and the time step used is dt=0.0001 in the reduced units defined by ϵ and σ. The number of integration steps of the simulation depends on the time the system takes to attain equilibrium, ranging from $1.5\times 10^{8}$ to $5\times 10^{8}$ steps depending on the conditions considered. Such long equilibration times require long computational times for each of the thermodynamic states considered, limiting the number of cases that we can explore. The performance of the simulations required about 2500 h of computation on the GPUs.

## 3. Thermodynamical Model

To understand the results from numerical simulations we built a simple thermodynamical model which allows us to predict the type of aggregate distribution that we might expect in equilibrium. The model is based on self-assembly theory [26,27] and a simplified account of the depletion interaction between rods which emerges due to the presence of depletant.

The effective depletion interaction potential between two colloidal particles is, to a good approximation, given by [1,7,28]

(1) $$W\left(h\right)=-c_{D}k_{B}TV_{ov}\;,$$

where *T* the temperature, $c_{D}$ the concentration of the depletant particles, and $V_{ov}$ the overlap volume of the regions around the colloidal particles excluded to the presence of depletant. The range of the interaction is given by the size of the depletant particles *D*, which is much smaller than the length and width of the aggregating rods.

For a slender body with length *L* and radius *R* (R/L≪1), the side-to-side interactions dominate over tip-to-tip interactions. This is because the reduction of the volume excluded to the presence of depletant is much smaller in the latter than in the former situation (to order R/L). As a consequence, aggregates are formed on the plane perpendicular to the axis of the colloids. For long rods with a square section of side *a*, we can easily quantify the interaction potential (Equation (1)) as the side-to-side overlap volume of two parallel rods separated a distance *d* is given by aL(D−d) if d<D, and 0 if d>D.

Due to the short range of the depletion interaction (∼D≪a≪L), we can consider that the total interaction energy of an aggregate is proportional to the number of contacts *m* between rods within the aggregates, $U=-m\epsilon_{0}$. Here, $\epsilon_{0}=c_{D}k_{B}TaLDf/2$ is the average energy of a single bond, with *f* a numerical factor which must account for the fact that there is a distribution of different degree of overlap between rods.

The equilibrium distribution of the number of aggregates formed due to depletion forces between rods can be understood using self-assembly theory. This approach was developed to describe the aggregation of amphiphiles [26,27] and it has also been used to theoretically account for the self-assembly of magnetic colloids [29,30]. In this formulation, aggregates with different size or internal energy are considered as different species which in equilibrium can reversibly convert into one another.

In dilute solutions of rods in which we can assume ideal mixing, the chemical potential of a rod in an aggregate with *n* rods and *m* contacts is given by [27]

(2) $$\mu_{n,m}=\mu^{0}+\frac{1}{n}\left[k_{B}T\ln\left(\frac{\phi_{n,m}}{n}\right)-m\epsilon_{0}\right]\;,$$

where $\phi_{n,m}=c_{n,m}/c_{0}$ is the concentration of rods in aggregates with *n* rods and *m* contacts over a reference concentration $c_{0}$. The conditions of thermodynamic equilibrium for the different species ($\mu_{n,m}=\mu_{1}\;,\forall n,m$) and the conservation of the total rod concentration $\phi_{R}=\sum_{n=1}^{\infty }\sum_{m=m_{n,min}}^{m_{n,max}}\phi_{n,m}$ completely define the state of the system, leading to a geometric distribution in the number of monomers per aggregate [27,29,30]. Thus, the probability of finding an aggregate of *n* rods is given by

(3) $$P\left(n\right)∼\left(e^{1/\langle n\rangle }-1\right)e^{-n/\langle n\rangle }\;.$$

Here, 〈n〉 is the average number of rods per aggregate, which depends on the ratio $\epsilon_{0}/k_{B}T$ and the total rod concentration $\phi_{R}$. In particular, self-assembly theory predicts that in equilibrium the average number of rods per aggregate should scale as $\langle n\rangle ∼\sqrt{\phi_{R}}$ for 〈n〉≫1. Note that due to the entropic origin of the depletion interaction, the distribution of aggregates (Equation (3)) does not depend on the temperature of the system.

## 4. Results and Discussion

We identify the formation of aggregates as the simulations advance. Aggregation of rods is observed to increase as the depletant concentration increases, in agreement with Equation (1). A significant aggregation of rods is observed for systems with concentrations of depletant high enough to induce depletion interactions able to overcome thermal fluctuations. The colloidal rods tend to assemble forming bundles, with preferential side-to-side over tip-to-tip or tip-to-side aggregation. In Figure 1b, we show representative morphologies obtained in our simulations. In all cases, the most likely structure is the single rod (Figure 1b1). Aggregation of pairs of rods occur almost exclusively through side-to-side interactions. Note that due to the existence of grooves on the side surfaces of the rods, the assembly between two rods takes place out-of-registry (Figure 1b2). For cases with high depletant concentration, larger bundles are formed; they still maintain, however, preference for side-to-side formations (Figure 1b3,b4).

**Figure 2:**
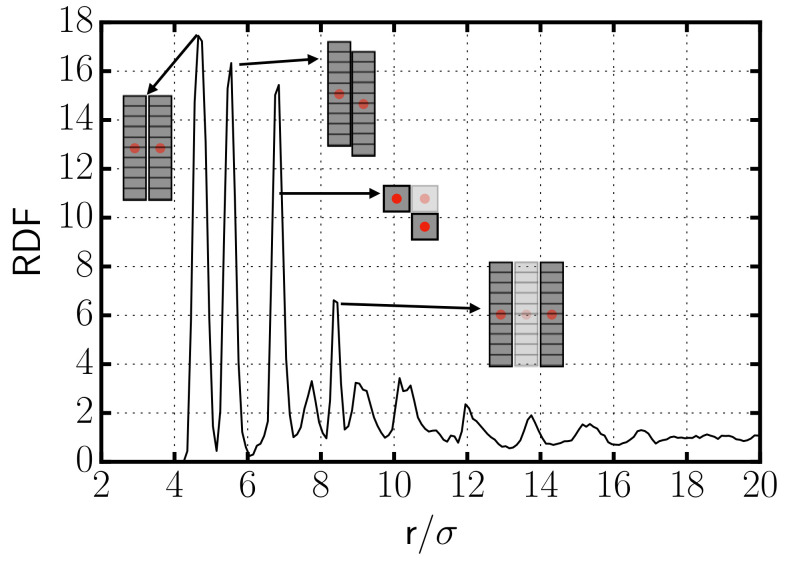
Radial distribution function for the centers of the rods for the case with $c_{D}=0.129\sigma^{-3}$. The pictures in the interior identify the configurations which originate the different peaks in the RDF. Note that while the configurations corresponding to the first, second, and fourth peaks are represented using a side view of the rods, the configuration leading to the third peak is represented using a top view of the rods.

In Figure 2, we show the radial distribution function (RDF) of the centers of the rods of a typical simulation in which aggregates are formed. The RDF exhibits a very prominent peak at the distance of minimum approach, r≈a, indicating the existence of aggregation. Due to the way the rods are constructed, with parallel planes of spheres, there is also a prominent peak corresponding to side-to-side aggregation of two rods whose sides are displaced by one plane (see Figure 2). The third peak corresponds to side-to-side arrangements of at least three rods forming an L-shape in the plane perpendicular to the axis of the rods (see picture in Figure 2). The last well-distinguishable peak is originated by side-to-side aggregates with at least three rods on a row. This RDF evidences the formation of aggregates with preference to side-to-side over tip-to-tip interactions between the rods.

To characterize the formation of aggregates, we calculate the average number of discs per aggregate as a function of simulation time, n(t), from the trajectories of the simulations. We consider that two rods belong to the same aggregate if at least 10 of their constituent beads (there are 36 on the long sides and 25 on each tip) are within a distance of 1.5σ. This distance corresponds to the first minimum of the radial distribution function of the beads which define the shape of the rods. We checked that the results that we obtain do not depend qualitatively on the exact values that we use to define the aggregation criterion, within a reasonable range.

**Figure 3:**
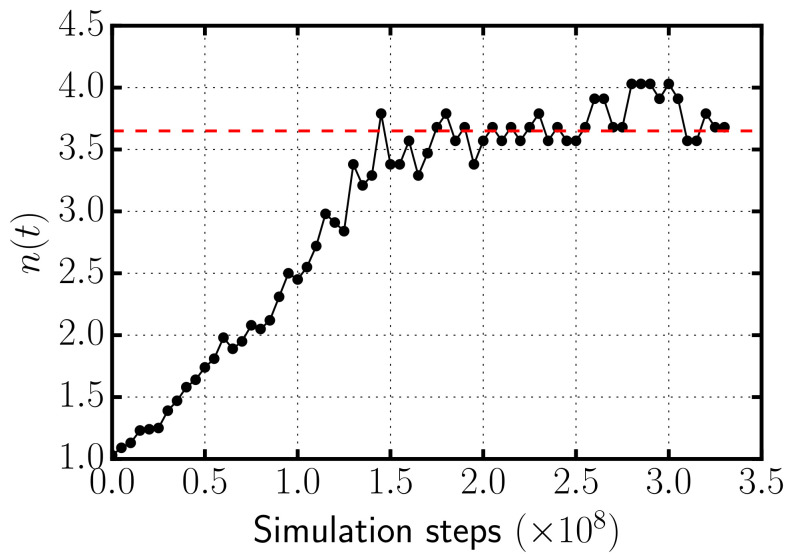
Black dotted line: Average number of rods per aggregate as a function of simulation time for the case with $c_{D}=0.118\sigma^{-3}$. The dashed red line indicates the average number that is computed taking the average over the frames once the system has reached equilibrium.

In Figure 3, we represent the average number of rods per aggregate as a function of simulation time for the case with depletant concentration $c_{D}=0.118\sigma^{-3}$. As the simulation progresses, depletion forces induce the self-assembly of rods into clusters of increasing size until a plateau is reached in which the formation and breaking up of bundles are compensated. Once n(t) reaches a plateau, the system is in thermodynamic equilibrium and average quantities are collected. We checked that the potential, translational, and rotational kinetic energies reach a plateau in equilibrium, and that the translational and rotational kinetic energies satisfy the equipartition theorem.

**Figure 4:**
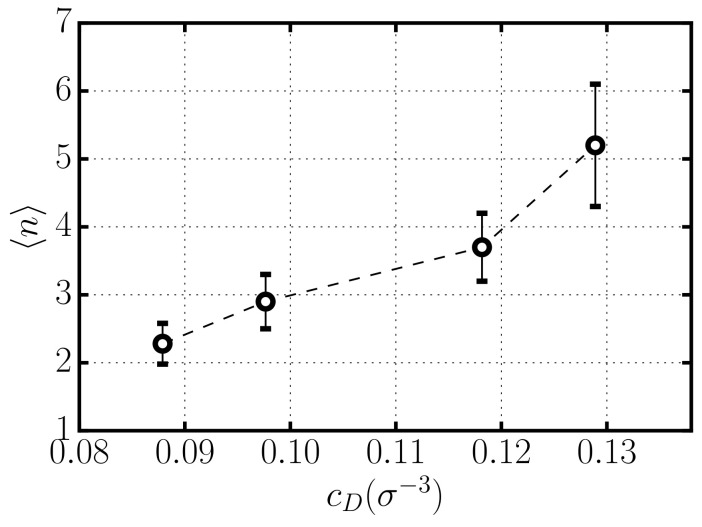
Number of rods per aggregate in equilibrium for systems with different depletant concentration $c_{D}$.

In Figure 4, we show the average number of rods per aggregate in equilibrium as a function of the concentration of depletant $c_{D}$. Our results show a substantial increase of aggregation with $c_{D}$. In fact, the strength of the attractive depletion interaction between rods increases linearly with depletant concentration (see Equation (1), inducing the formation of larger aggregates. This result is consistent with the expectation from thermodynamic models of self-assembly [27,30], which predict an exponential dependence of aggregation on the strength of the mutual attractive interaction.

**Figure 5:**
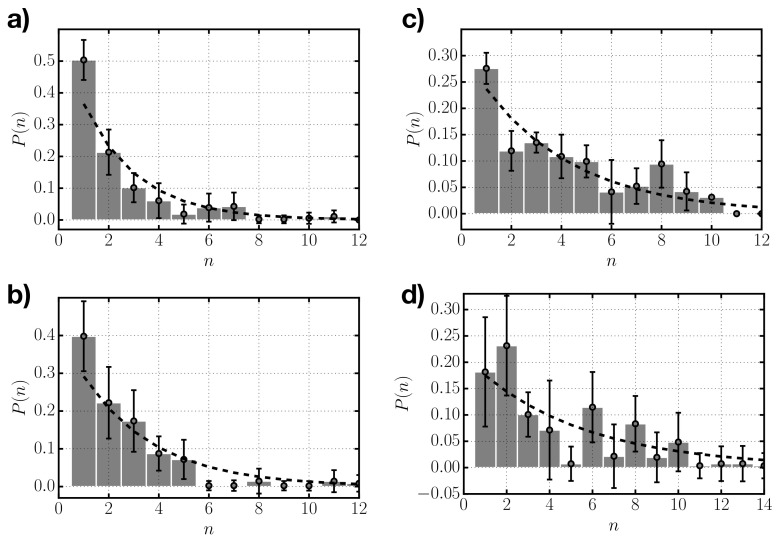
Frequency of occurrence P(n) versus aggregate size *n* as obtained from numerical simulations (gray bars) for systems with depletant concentrations: (**a**) $c_{D}=0.088\sigma^{-3}$, (**b**) $c_{D}=0.098\sigma^{-3}$, (**c**) $c_{D}=0.118\sigma^{-3}$, and (**d**) $c_{D}=0.129\sigma^{-3}$. Superimposed to the results from numerical simulations we plot the geometric distribution given by Equation (3) using the average values 〈n〉 obtained from numerical simulations (dashed black line).

From our simulations we also have access to the size distribution of clusters. We analyzed the average size distribution of the different systems considered once they reach thermodynamic equilibrium. In Figure 5, we represent the average probability of finding a cluster of a given size *n* (i.e., composed of *n* rods) for systems with different depletant concentration. For the lowest value of $c_{D}$ (Figure 5a), the distribution indicates a large proportion of single rods (≈50%) and decays monotonically with cluster size. As the depletant concentration increases, the distribution becomes flatter, with less dominance of single monomers and more weight of larger sizes. The distributions obtained from simulation are subject to significant fluctuations due to the reduced size of the systems considered (with 125 rods), especially for the highest depletant concentration, $c_{D}=0.129\sigma^{-3}$, in which there is a wider range of statistically significant possible aggregates. In Figure 5, we also show the theoretical geometrical distributions (Equation (3)), which correspond to the values of 〈n〉 for each $c_{D}$ obtained from numerical simulations. Despite the statistical fluctuations in the distributions obtained from simulation, the agreement with the geometrical distributions is reasonable.

The predominance of side-to-side aggregation and the type of size distribution of clusters should be independent of the cross section of the rods as long as their aspect ratio is small enough (R/L≪1). In contrast, the strength of the depletion interaction can be affected by the shape of the rods’s cross section, as it determines the overlap volume excluded to the presence of depletant (see Equation (1). Consequently, the concentration of depletant which marks the inset of strong aggregation can be greatly dependent on the shape of the rods’ cross section.

## 5. Conclusions

We investigated the self-assembly of slender rods in solution due to the effective depletion interaction that emerges due to the presence of depletant particles. We performed computer simulations at fixed temperature and volume of a simple model composed of rod-like particles and smaller spherical depletant particles which interact solely through repulsive interactions. In this system, depletion forces between rods emerge naturally for certain values of the concentration of rods and depletant, inducing the aggregation of rods into clusters.

We verified that, as expected, clusters form preferentially due to side-to-side interactions, growing in the plane perpendicular to the axes of the rods. Aggregation due to tip-to-tip interactions is anecdotal. We characterized the growth of these clusters over time, observing a monotonical increase in the average size of aggregates up to a stable plateau when the system reaches the equilibrium state.

The equilibrium state was analyzed for systems with different depletant concentration. We observe that the aggregates’ average size increases rapidly with depletant concentration, as expected. Indeed, mean-field thermodynamic models of aggregation anticipate an exponential increase of the average aggregate size with the strength of the interaction governing self-assembly. In our case, the formation of clusters is led by the depletion interaction, which increases linearly with depletant concentration. We also obtained the size distributions obtained in equilibrium from our numerical simulations. We show that the numerical results are consistent with a geometrical distribution predicted by basic thermodynamic models of self-assembly.

## Supplementary Materials

The following are available online at https://www.mdpi.com/1099-4300/22/10/1114/s1: python script to set up the system and perform the simulations using HOOMD-blue 2.2.3 reported in this article.
