# Supplementary material for: Self-Assembly of Microscopic Rods Due to Depletion Interaction
Source: Entropy (Basel). 2020 Oct 1;22(10):1114. doi: 10.3390/e22101114 (PMC7597238; doi:10.3390/e22101114)
Supplement: Supplementary file 1 [file entropy-22-01114-s001.zip › Supplemental_Material_submit/Supplemental_material.pdf]

SUPPLEMENTAL MATERIAL: “Self-assembly of microscopic rods due to depletion interaction”

Carles Calero and Ignacio Pagonabarraga

We attach an example of a simulation script called “script_simulation.py” to be run on HOOMD-Blue. The script defines the simulation box, the objects (rods and spherical depletant particles) to be simulated and their interactions. The simulation method and the temperature of the thermostat are also provided. The script also initializes the positions of all the particles in a lattice in a large simulation box. From such positions the script contains two runs. First, there is an initial compression of the cubic simulation box until the desired particle density is reached. The second run corresponds to the start of the simulations reported in the manuscript, which uses the last frame of the compression run as initial condition.
